# Antiretroviral regimens sparing agents from the nucleoside(tide) reverse transcriptase inhibitor class: a review of the recent literature

**DOI:** 10.1186/1742-6405-10-33

**Published:** 2013-12-13

**Authors:** Amit C Achhra, Mark A Boyd

**Affiliations:** 1 The Kirby Institute, University of New South Wales, Sydney, Australia

**Keywords:** HAART, Toxicity, NRTI, NNRTI, NRTI-sparing, Class-sparing, HIV, Dual therapy, Raltegravir, Maraviroc

## Abstract

The nucleoside(tide) reverse transcriptase inhibitors (NRTIs) have traditionally been an important ‘back-bone’ of an antiretroviral therapy (ART) regimen. However all agents have been associated with both short- and long-term toxicity. There have also been concerns regarding the efficacy and safety of a treatment sequencing strategy in which those with past exposure and/or resistance to one or more NRTIs are re-exposed to ‘recycled’ NRTIs in subsequent ART regimens. Newer, potent and possible safer, agents from various ART classes continue to become available. There has therefore been growing interest in evaluating NRTI-sparing regimens. In this review, we examined studies of NRTI-sparing regimens in adult HIV-positive patients with varying degrees of ART experience. We found that in treatment experienced patients currently on a failing regimen with detectable viral load, there now exists a robust evidence for the use of NRTI-sparing regimens including raltegravir with a boosted-protease inhibitor with or without a third agent. In those on a virologically suppressive regimen switching to a NRTI-sparing regimen or in those ART-naïve patients initiating an NRTI-sparing regimen, evidence is sparse and largely comes from small exploratory trials or observational studies. Overall, these studies suggest that caution needs to be exercised in carefully selecting the right candidate and agents, especially in the context of a dual-therapy regimen, to minimise the risks of virological failure. There is residual toxicity conferred by the ritonavir boost in protease-inhibitor containing NRTI-sparing regimens. Fully-powered studies are needed to explore the place of N (t)RTI-sparing regimens in the sequencing of ART. Additionally research is required to explore how to minimise the adverse effects associated with ritonavir-based pharmacoenhancement.

## Introduction

The nucleoside(tide) reverse transcriptase inhibitor (NRTI) class has formed the ‘back-bone’ of antiretroviral therapy (ART) regimens since the early ART era. All major treatment guidelines currently recommend the selection of 2 NRTIs and a third agent from a separate class in the initial management of ART naïve HIV-positive patients; some guidelines suggest recycling NRTI agents as far as possible in ART-experienced HIV-positive patients experiencing virological failure [1-4]. However, concerns about long-term toxicities and cross--resistance within the NRTI class combined with the continuing development of newer, seemingly safer agents in several independent classes of drugs has led to a growing interest in the potential use of feasible, innovative and appealing NRTI-sparing options.

The main purpose of this review is to explore the current state of our knowledge on the efficacy and safety of NRTI class-sparing antiretroviral regimens used in the management of adult HIV-positive patients. The review is restricted to studies conducted on adult HIV-1 mono-infected individuals published in the English language. The review has a greater focus on studies published since 2006 (so as to ensure relevance to contemporary practice). We included clinical trials as well as non-randomised small clinical studies, both published and unpublished (conference presentations).

### Rationale for NRTI-sparing regimens

The thymidine analogue NRTIs, such as zidovudine and in particular stavudine (d4T) have been associated with serious mitochondrial toxicity which has been linked to lipoatrophy. In addition, zidovudine has been associated with anaemia and didanosine with peripheral neuropathy. As a consequence these agents are no longer recommended as preferred components of therapy; in the case of stavudine it has been recommended that it’s use be phased out, even in low- and middle-income countries [2]. However, one of the most important push factors for the assessment of NRTI-sparing regimens has been a growing understanding of the long-term toxicity profile of even the newer, relatively safe NRTIs in common use such as tenofovir and abacavir. Tenofovir has been associated with nephrotoxicity, including acute and chronic renal failure, proximal tubular dysfunction, nephrogenic diabetes insipidus and nephrotic syndrome [1,4,5]. In a large EuroSIDA cohort study, tenofovir use was associated with a 20% increased risk of chronic kidney disease [6]. Further, clinical trials and observational studies have consistently demonstrated decline in bone-mineral density (BMD) of about 2-10% in the short-term attributable to the use of tenofovir [7,8]. Cumulative use of tenofovir has been associated with a higher risk of osteoporotic fracture [9]. Finally, one observational study attributed increased risk of heart failure to the tenofovir use, although this observation has not been replicated [10]. Abacavir, another commonly used NRTI has been implicated in the risk of myocardial infarction by some large cohort studies and a clinical trial [10-12]. However, the evidence is not definitive as this association has not been seen in other studies [13]. Nevertheless, it has generated considerable anxiety in the provider community, especially for its use in patients with a high cardiovascular risk. Further, abacavir is associated with the risk of severe hypersensitivity reactions which can only be eliminated by screening out those carrying the HLA-B57*01 allele [1,14].

One of the main concerns prompting studies on NRTI-sparing regimens in failing patients has been that of possible reduced potency of NRTI agents due to current or archived nucleoside mutations such as thymidine analogue associated mutations (TAMs). As a result, if NRTIs are being recycled in such patients, they could potentially be exposed to a regimen with <2 or 3 fully-active agents. This is especially of concern in resource limited settings where NRTIs are recommended for recycling following treatment failure identified through immunological or virological failure. This surrogate means of identifying virological failure is associated with substantial degrees of resistance which may significantly compromise the action of recycled NRTIs in future ART regimens [15]. Moreover, access to agents beyond NRTIs and the ritonavir-boosted protease inhibitors atazanavir, lopinavir and darunavir is limited [2]. Switching a specific anchor agent to one with a lower genetic barrier (e.g. LPV/r replaced by raltegravir [16] or PI replaced with another NRTI [17]) with a compromised NRTI backbone could result in higher rates of treatment failure. Finally, recycling NRTIs might mean exposing patients to long term NRTI agents which may result in unanticipated, cumulative adverse effects.

Several newer agents from various classes with a good safety profile to date continue to become available. These include new integrase inhibitors, newer non-nucleoside reverse transcriptase inhibotors (NNRTIs, e.g. etravirine and rilpivirine), the CCR5 attachment inhibitor maraviroc and the PIs lopinavir, darunavir and atazanavir. It is therefore possible to construct regimens containing 2-3 fully-active agents which are NRTI-sparing.

We next discuss NRTI-sparing regimens that have been evaluated in each of the three main types of patient populations: treatment-experienced failing patients where most of the evidence is concentrated; virologically suppressed patients; and ART-naïve patients.

### NRTI-sparing regimens in treatment-experienced failing patients

Recent large clinical trials have evaluated NRTI-sparing regimens in treatment experienced patients experiencing virological failure (key studies summarised in Table 1[18-25]). The regimen most of interest has been that of a boosted-PI with raltegravir, and/or maraviroc and/or etravirine.

**Table 1:** Key recent studies of NRTI-sparing regimens in treatment experienced patients

**Author, name of the trial, if any/year/published?**	**Design**	**Comparison**	**N**	**Follow-up (weeks)**	**Results**
Boyd et al. [18], SECOND-LINE/2013/Yes	Phase-3/4 RCT, non-inferiority	(i) LPV/r + RAL vs. (ii) LPV/r + recycled NRTIs in those failing 1^st^ line NNRTI-based ART	541 (271 vs. 270)	48	-Arm (i) non-inferior to arm (ii) for virological outcome
					-No major differences in serious adverse events
					-Greater decline in BMD in arm (ii) [25]
Paton et al. [19], EARNEST/2013/No	Phase-3/4 RCT, non-inferiority	(i) LPV/r + RAL	(i) 433; (ii) 418; (iii) 426	96	-Arm (i) non-inferior to arm (iii) for a composite of virological and clinical endpoint
		(ii) LPV/r monotherapy after induction with LPV/r + RAL			
		(iii) LPV/r + recycled NRTIs (control) in those failing 1^st^ line NNRTI-based ART			-Arm (ii) inferior to other arms for virological outcome and higher LPV/r resistance
					-No differences in grade 3/4 events
Tashima et al. [20], OPTIONS/2013/No	Phase-3/4 RCT, non-inferiority	(i) NRTI-omitting optimised regimen	(i) 179; (ii) 181	48	-Similar virlogical outcomes in both arms
		(ii) NRTI-including optimised regimen in triple-class experienced failing patients			-No differences in grade 3/4 events
					-Higher mortality in arm (ii).
Ruane et al. [24], INROADS/2013/No	Single-arm exploratory phase-2b trial	DRV/r + ETV in failing patients (78%) or ART naïve patients with transmitted resistance (22%)	54 (75% completed the study)	48	-100% of ART naïve and 87% of failing patients achieved virological success.
					-2 patients developed ETV mutations and none had DRV mutations.
Imaz et al. [21]/2011/Yes	Observational	Salvage regimen of at least three active agents from DRV, ETV, RAL and MVC, with or without NRTIs	122	48	-78% virologically suppressed (equal in both arms)
					-Higher baseline viral load associated with worse outcomes.
Nozza et al. [22]/2011/Yes	Observational	Salvage regimen of RAL + MVC + ETV	28	96	-96% virologically suppressed (<50 copies/ml)
Florence et al. [23]/2010/Yes	Observational	Salvage regimen of ETV + optimised regimen, 40% without NRTIs	941	24	-70% and 90% had viral load <50 and 400 copies/mL respectively.

The SECOND-LINE study (n=541) [18] evaluated ritonavir-boosted lopinavir (LPV/r) with raltegravir vs. LPV/r + NRTIs (the WHO standard of care of recycling NRTIs in HIV-positive patients with demonstrable virological failure of first-line NNRTI-based ART), with no previous PI or integrase inhibitor exposure. The use of resistance testing at randomization was optional. Of the 492 patients with resistance tests available, 89% had ≥1 NRTI mutations and 60% had M184V with ≥ NRTI mutations at baseline. Patients were randomised (stratified by baseline HIV RNA ≤ or > 100,000 copies/mL) to receive LPV/r with raltegravir or LPV/r with physician-chosen NRTIs. At 48 weeks, the NRTI-sparing raltegravir arm was non-inferior to the NRTI-containing control arm for the primary endpoint of virological control <200 copies/mL with an overall 81.7% virological response rate. The NRTI control-arm resulted in a 2-4% greater decline in BMD compared to the NRTI-sparing arm (likely due to patients switching to tenofovir) [25]. On the other hand the raltegravir arm was associated with a greater degree of hypercholesterolaemia. Despite this there was no difference in the resultant total: HDL cholesterol ratio. Finally, emergence of PI mutations was negligible in those experiencing virological failure, attesting to the high genetic barrier of LPV/r under conditions of frequent (3 monthly) virological monitoring.

The EARNEST study [19] was similarly designed to SECOND-LINE and performed in an identical patient population (with an additional arm of LPV/r monotherapy after a 12 week induction combined with raltegravir). After 96 weeks of follow-up the investigators found no difference between the raltegravir and NRTI-arms in the study’s primary composite endpoint of “good disease control” (i.e. alive with no WHO stage 4 disease, CD4 >250/mm^3^ and viral load <10,000 or >10,000 copies/mL with no PI mutations), as well as virological outcome (viral load <200 or <50 copies/mL). The investigators also found no difference in grade 3/4 events at 96-weeks follow-up. Of note, the monotherapy arm was found to be virologically inferior to the control and LPV/r plus raltegravir arms.

The OPTIONS study [20] was conducted on a set of treatment experienced patients failing a PI-based regimen and with past exposure to NRTIs and NNRTIs (n=360). Patients were randomised to either NRTI-including or NRTI-excluding optimised regimen arms containing >2 fully active agents (not including NRTIs). In the NRTI-sparing arm, the most common regimens were raltegravir with boosted-darunavir (DRV/r) and either etravirine (56%), maraviroc (14%) or both (9%). At 1 year of follow-up, virological suppression rates were similar in both arms. Of note, the study only had the power to find a non-inferiority margin of 15%. Finally, though there were no major differences in the grade 3/4 safety outcomes the NRTI-arm experienced greater mortality. It is unclear if causes of deaths were NRTI-related.

A few smaller observational studies confirm these findings in routine clinic settings (Table 1). In one study of 122 patients with prior triple-class failure, the regimen of raltegravir with DRV/r and maraviroc or etravirine demonstrated similar virological efficacy (>75%) compared to those using NRTI agents at 48 weeks [21].

Collectively, these studies confirm that in virologically failing patients, the strategy of selecting 2 or 3 fully-active pharmacologically compatible agents is a feasible strategy. Of note, all of these studies have included a boosted-PI, and because of their high genetic barrier to resistance, the data should not be extrapolated to other 2-class regimens. Also, recycled NRTI agents seem to retain efficacy (i.e. non-inferior to the comparator arms), which is reassuring in settings where newer agents are not yet widely available.

### NRTI-sparing regimens in patients receiving suppressive ART

Switching to a NRTI-sparing regimen in a virologically suppressed patient needs consideration of several factors. These include the treatment history, archived mutations, duration of viral suppression, expected level of adherence, the choice of agents as well as the patient motivation to switch from a stable regimen. Large, well powered trials in such patients are few and difficult to recruit, although several smaller studies have identified promising regimens. Key studies on this patient population are summarised in Table 2[26-36].

**Table 2:** Key recent studies of switch to NRTI-sparing regimens in virologically suppressed patients on standard ART

**Author, name of the trial, if any/year/published?**	**Design**	**Comparison**	**N**	**Follow-up (weeks)**	**Results**
Monteiro et al. [36]/2013/Yes	Observational	RAL + ETV	25	48	-91% virologically suppressed in per-protocol analysis
					-Lipids improved
Ward et al. [26]/2013/No	Observational	Switching for toxicity concerns to a RAL + 1 or 2 agents, most commonly on RAL + ATV/r with or without ETV or MVC	62	168	-92% virologically suppressed;
					-3 of 15 on dual therapy had to add third agent for low-level viremia
Calin et al. [27]/2013/No	Observational	Switching to RAL + ETV regimen	91	48	-93% had viral load <50 copies/mL
					-4/5 with virological failures had past NNRTI mutations
					-3 patients had RAL mutations
Katlama et al. [28], ROCnRAL/2013/No	Single-arm exploratory trial	R5-trophic suppressed patients switched to MVC + RAL	41	48	-Failure in 11.4%
					-RAL mutations in 3/5 patients who failed
					-1/5 had R5 to ×4 virus switch
Cotte et al. [29], No Nuc No Boost/2013/No	Single-arm exploratory trial	MVC + RAL	10	48	-No virological failures (>50 copies/mL)
					-No serious adverse event
Burgos et al. [30]/2012/No	Observational	Switching for toxicity concerns to a PI/r + 2^nd^ agent, many with no NRTI	131	56	- > 90% virologically suppressed.
Ofotokun et al. [31], KITE/2012/No	Exploratory pilot trial	(i) LPV/r + RAL; (ii) standard ART	60	48	-92% in arm (i) and 88% in arm (ii) with viral load <50 copies/mL;
					-Higher triglycerides in arm (i)
					-No difference in BMD or body composition.
Carey et al. [32], SPARTA/2012/Yes	Pilot cross-over RCT	Patients receiving ATV/r randomized to: (i) ATV/r (300/100 mg respectively once daily) + RAL (800 mg once daily)	25	76% in follow-up for 48 weeks	-Both agents pharmacologically compatible.
		(ii) ATV (300 mg twice daily) + RAL (400 mg twice daily)			-All patients remained virologically suppressed
Cordery et al. [35]/2010/Yes	Observational	RAL + ATV (unboosted)	20	72	-Only 1 (5%) failure
Allavena et al. [33]/2009/Yes	Observational	Switching for toxicity concerns to a PI/r + RAL.	29	48	-100% virologically suppressed
Fischl et al. [34]/2007/Yes	RCT, not fully powered	(i) LPV/r + EFV;	236	96	-Arm (i): shorter time to failure or discontinuation;
		(ii) EFV + NRTIs			
					-Arm (i): greater increase in triglycerides

In the ACTG 5116 trial [34], virologically suppressed patients receiving a standard PI- or NNRTI-based ART regimen were randomised to efavirenz with LPV/r or efavirenz with NRTIs (standard ART arm). Though not a fully powered trial, the NRTI-sparing arm performed poorly due largely to higher rates of discontinuation and toxicity as well as dyslipidemia. This study suggests that the choice of agents will be important in constructing such regimens as they will need to provide clear evidence of greater safety before convincing ART providers and/or patients to consider a switch.

In a more recent small exploratory study (the KITE trial [31]), virologically suppressed patients with no history of failure to PI-based regimens were randomised to standard ART (n=20) or LPV/r with raltegravir (n=40). At 42 weeks, there were no differences in virological suppression rates (90-92%) and no treatment limiting side effects, although there was a trend for higher triglycerides in the NRTI-sparing arm. Another similar exploratory single-arm trial [33] (n=29) reported similar results with raltegravir plus a boosted-PI (mainly darunavir) regimen with no serious dyslipidaemia at 24 weeks.

Since most studies on NRTI-sparing regimens have included a boosted-PI regimen, residual toxicity concerns with ritonavir as the booster (such as gastrointestinal upset and dyslipidemia) have been seen as a relative disadvantage. With several new agents now available, it is now possible to conceive regimens which exclude not only NRTIs but also ritonavir, as well as first-generation NNRTIs such as efavirenz (also associated with dyslipidaemia and neurotoxicity) or nevirapine. This strategy is especially attractive in virologically suppressed patients with minimal treatment experience, where good adherence can be presumed and the possible adverse impact of high viral load on regimen efficacy is less of a concern.

In one recent cohort study on treatment experienced, virologically suppressed patients switching to dual therapy of etravirine with raltegravir, 91% maintained virological suppression at 48 weeks [36]. In another exploratory observational study of 62 patients (the majority with HIV RNA <400 copies/mL) switching to NRTI-sparing regimens, 92% had undetectable viral loads at 42 months [26]. The most common regimens used were: raltegravir, unboosted atazanavir, and maraviroc (33%); raltegravir and unboosted atazanavir (22%); raltegravir, maraviroc, and etravirine (13%). Of note, 3 out of 15 people receiving a two drug regimen demonstrated low-level viremia (i.e. >50 but <200 copies/mL), the clinical meaning of which is debated. In another study, called No Nuc No Boost [29], ART-naïve R5 tropic patients with high CD4 counts and baseline viral load <10^3^ log copies/mL were first treated with tenofovir/emtricitabine, raltegravir and maraviroc for six months after which those with plasma viral load <50 copies/mL at 24 weeks were continued on a two-drug combination of raltegravir and maraviroc for a further 24 weeks. All patients retained viral load <50 copies/mL on dual therapy. However, in another similar study (RocNRaL) on more treatment experienced patients, this dual regimen demonstrated lower efficacy [28]. Collectively, these studies suggest that while such unconventional regimens are promising in carefully selected patients, caution must be exercised when using dual therapy regimens without the support of a high genetic barrier to the selection of resistance like LPV/r or DRV/r.

#### Boosted-PI monotherapy

Given the high genetic barrier of boosted PIs, several large studies have evaluated this simplification strategy. Boosted-PI monotherapy trials have been extensively reviewed previously [37,38] and are therefore not covered in detail here. In the 96-week trial of LPV/r monotherapy in virologically suppressed patients receiving a boosted-PI + NRTI regimen with no history of failure (OK trial), although the virological success rate was similar, the monotherapy arm had a significantly higher rate of low-level viremia (12%) necessitating reinduction with NRTIs [39]. A similar trial with DRV/r monotherapy (MONET) [40] also demonstrated a high virological success rate with DRV/r however the monotherapy arm had a higher rate of low-level viremia. Overall, although the virological response rates in LPV/r or DRV/r mono-therapy arms are high and PI mutations are rare in the setting of regular virological monitoring, there appears to be a greater risk of consistent low-level viremia which may herald virological failure in the monotherapy arms. In the fully powered EARNEST study [19] described above, the PI monotherapy arm (combined for the first 12 weeks of treatment with RAL 400 mg bid) was found to be significantly inferior to the standard ART arm. The authors concluded that such a strategy is unsuitable for a public health approach. Presently, none of the major treatment guidelines recommend PI monotherapy because of concerns regarding efficacy [1-4].

### NRTI-sparing regimens in ART naïve patients

Large, fully powered trials evaluating NRTI sparing regimens in ART naïve patients are few and most smaller studies have suggested that care must be exercised in selecting both suitable agents and candidates for such regimens. Key studies are summarised in Table 3[41-48].

**Table 3:** Key recent studies of switch to NRTI-sparing first-line ART regimens in ART naïve patients

**Author, name of the trial, if any/year/published?**	**Design**	**Comparison**	**N**	**Follow-up (weeks)**	**Results**
Mills et al. [41], A4001078/2013/Yes	RCT, phase-2b pilot	(i) MVC + ATV/r	121	48	-75% in arm (i) and 84% in arm (ii) had viral load <50 copies/mL.
		(ii) TDF + FTC + ATV/r			
					-More hyperbilirubenmia in arm (i)
					-Nine in arm (i) and 3 in arm (ii) had low-level viremia after virological suppression
Reynes et al. [42], PROGRESS/2013/Yes	RCT pilot study	(i) LPV/r + RAL	(i) 101; (ii) 105	96	-66.3% in arm (i) and 68.6% in arm (ii) responded by FDA-TLOVR
		(ii) LPV/r + TDF + FTC			
					-Better body comp in arm (i)
					-Greater decline in eGFR in arm (ii)
Kozal et al. [47], SPARTAN/2012/Yes	RCT pilot study	(i) ATV + RAL	(i) 63; (ii) 31	24	-74.6% in arm (i) and 63.3% in arm (ii) had viral load <50 copies/mL
		(ii) ATV/r + TDF + FTC			
					-4/6 failures in arm (i) had RAL mutations.
					-20% incidence of grade-4 hyperbilirubenimia in arm (i).
Taiwo et al. [43,48], MIDAS/2013/Yes	Single-arm pilot	MVC + DRV/r	25	96	-Viral load < 50 copies/mL: 8.3% and 10% at week 48 and 96, respectively.
					-Virological failures mainly explained be high baseline viral load >100000 copies/mL
Bedimo R et al. [44], RADAR/2011/No	RCT pilot	(i) RAL + DRV/r	80	24	-86% in arm (i) and 87% in arm (ii) had viral load <50 copies/mL
		(ii) DRV/r + TDF + FTC			
Taiwo et al. [45], ACTG5262/2011/Yes	Single-arm pilot	DRV/r + RAL	112	48	-26% with viral load > 50 copies/mL, majority with low-level viremia (<200 copies/mL)
					-Baseline viral load >100000 copies/mL strongly associated with failure
Riddler et al. [46], ACTG5142/2008/Yes	RCT	(i) EFV + NRTIs	(i) 250	96	-89%, 77% and 83% had viral load <50 copies/mL in arms (i), (ii) and (iii) respectively
		(ii) LPV/r + NRTIs	(ii) 253		
		(iii) LPV/r + EFV	(iii) 250		
					-No difference in time to toxic effects
					-At failure, resistance mutations more common in arm (iii)

NOTE for Tables 1, 2, 3: ATV/r = ritonavir boosted atazanavir, DRV/r = ritonavir boosted darunavir, LPV/r = ritonavir boosted lopinavir, RAL = raltegravir, ETV = etravirine, MVC = maraviroc, EFV = efavirenz, TDF = tenofovir, FTC = emtricitabine, NRTI = Nucleoside(tide) reverse transcriptase inhibitors, PI = protease inhibitors.

The ACTG 5142 trial randomised ART-naïve patients to standard ART arms with LPV/r or efavirenz, both given with investigator selected 2NRTIs and a NRTI-sparing arm of LPV/r and efavirenz [46]. Though the NRTI-sparing arm showed comparable virological efficacy overall at 96 weeks, it performed poorly in those with baseline viral load >100,000 copies/mL and the rate of discontinuation due to adverse events was significantly higher than other two arms. Of note, the dose of LPV/r was increased due to its interaction with efavirenz, which may have contributed to the additional toxicity in this arm.

More recent pilot studies have explored a dual therapy regimen of raltegravir with a boosted-PI. These studies suggest that a high baseline plasma viral load could adversely impact the success of the regimen in such patients. In a single arm study of 112 patients receiving raltegravir and DRV/r, virological failure (>50 copies/mL) was 26% at 48 weeks [45]. Most (21/28) of the patients with virological failure and all of those with integrase mutations had a baseline plasma viral load >100,000 copies/mL . Almost 50% of failures were that of low level viremia (>50 but <200 or <400 copies/mL). No PI mutations were detected. However, the similar fully-powered trial of DRV/r + RAL (NEAT protocol 001, see below) is ongoing and has not been stopped by Data Safety Monitoring Board. In the RADAR study (small randomised study of DRV/r with either raltegravir or NRTIs), response rates were poorer in the raltegravir arm which also experienced higher rates of dyslipidemia [44]. In the SPARTAN pilot study evaluating experimental unboosted atazanavir (300 mg) with raltegravir (vs. NRTI with boosted-atazanavir), most raltegravir resistance mutations occurred in those with baseline viral load >100,000 copies/mL [47]. Also, the rate of hyperbilirubinemia in the raltegravir arm was higher (possibly due to the higher dose of atazanavir used in that arm (300 mg bid) and the known pharmacoenhancement effect of RAL on ATV) [47]. In a randomised study of LPV/r with raltegravir or with NRTIs (PROGRESS trial), virological response did not differ by baseline viral load although there were only a few patients with high viral load at baseline [42]. Of note, in this study, surrogate renal and bone outcomes were significantly more favourable in the NRTI-sparing arm.

Overall, these studies suggest that while NRTI-sparing regimens are promising, caution needs to be exercised in the selection of patients. In particular, the impact of the baseline plasma viral load on regimen efficacy and the risk of baseline or archived resistance mutations for failure need further study. Dual-therapy regimens in general have resulted in mixed results. Well designed, appropriately powered randomised controlled trials are required to reach definitive conclusions. In a recent well-powered trial of LPV/r with lamivudine (a relatively safe NtRTI) vs. LPV/r+2 NRTIs, the dual therapy arm demonstrated non-inferior efficacy of >85% at 48 weeks regardless of baseline viral load, with fewer toxicity-related discontinuations [49]. This study indicates that carefully selected dual therapy could be a reasonable option even in ART-naïve patients. Of note, however, dyslipidemia tended to be more pronounced in the dual therapy arm, which may be due to the absence of tenofovir which is known to be associated with a favourable lipid profile [50,51]. On the other hand, the MODERN study – a well powered study of maraviroc + DRV/r vs. standard ART (n=791) was prematurely stopped at 48 weeks due to inferior performance of the dual-therapy arm (unpublished data) [52]. Ongoing studies such as A4001095 (DRV/r with maraviroc or NRTIs) (Trial Identifier: NCT01345630)and the NEAT protocol 001/ ANRS 143 (DRV/r with raltegravir or NRTIs) (Trial Identifier: NCT01066962) should provide a clearer understanding of the use of such regimens in ART-naïve patients.

### Limitations, conclusions and future directions

NRTI-sparing regimens appear to be promising and if established, will be an important step towards optimising ART to maximise patient safety. However, there are a few important points to consider. First, trials evaluating such regimens will need to show a clear benefit in terms of long-term safety so as to convince clinicians to move away from much more familiar NRTI-based regimens. Promising regimens such as the use of a boosted-PI with raltegravir have shown a higher risk of dyslipidemia in some studies likely attributable to ritonavir and possibly the absence of tenofovir. Future studies considering regimens without NRTIs, ritonavir and efavirenz (due to neuro-psychaitric adverse events) will be important in identifying safer, better tolerated ART regimens. A recent study by our group suggested that most HIV-positive patients will have such regimen options available to them regardless of their treatment experience [53].

In addition, most clinical trials follow participants up for 48 to 96 weeks, which, along with their enrolment number, is not of sufficient duration to demonstrate differences in hard clinical endpoints such as renal failure or fractures. Cohort studies will be instrumental in this regard. Also, most of the studies are small and unpublished. Better quality evidence is therefore clearly needed in this area.

Second, many of the studies on NRTI-sparing two drug regimens suggest that in selected patients (e.g. those with high baseline viral loads), the risk of virological failure and selection of resistance mutations to the non-PI component are a risk. This suggests that such regimens, especially ones without a boosted-PI support, should ideally be first evaluated in virologically suppressed patients.

Finally, many NRTI-sparing regimens are may require twice daily dosing frequency and ≥1-2 pills/day. This may impact the adherence (example selective lower adherence to the 2^nd^ dose of raltegravir in DRV/r + raltegravir regimen). However, both etravirine and maraviroc have pharmacokinetic and clinical data supporting once-daily dosing [41,54], and RAL is being studied in a 1200-mg once-daily formulation. Therefore, NRTI-sparing once-daily regimens could be possible in future.

In conclusion, there has now accumulated a sizeable evidence base that supports the use of NRTI-sparing regimens of 2–3 fully-active agents for HIV-positive patients currently on a failing ART regimen. For virologically suppressed patients, an NRTI-sparing regimen may be an option is some patients although further, more definitive studies are needed. Finally, the evidence is sparse for such regimens in the ART naïve patient population and more research is required to generate robust evidence. Future studies such as A4001095 and NEAT protocol 001/ ANRS 143 will be instrumental in informing the evidence base for such regimens.

## Competing interests

ACA has received Gilead Australia Fellowship for the year 2013. MB has received research grant support and honoraria for serving on advisory boards and educational presentations from AbbVie, Boehringer Ingelheim, Bristol Myers Squibb, Gilead Sciences, Janssen Pharmaceuticals, and Merck.

## Authors’ contributions

MAB conceived the idea. ACA and MAB both performed the literature search and planned the structure of the review. ACA conducted the review and wrote the first draft and subsequent changes to the manuscript. MAB provided key input to the contents and provided overall supervision of the project. All authors read and approved the final manuscript.
